# Novel Experimental Therapeutic Approaches in Glioma—New Hydrazide–Hydrazones as Chemical Agents Sensitizing Glioblastoma Cell Line to Radiotherapy

**DOI:** 10.3390/ijms27125459

**Published:** 2026-06-17

**Authors:** Dorota Natorska-Chomicka, Monika Gawrońska-Grzywacz, Paweł Patrejko, Mariola Herbet, Iwona Piątkowska-Chmiel, Magdalena Iwan, Jarosław Dudka, Łukasz Popiołek

**Affiliations:** 1 Chair and Department of Toxicology, Faculty of Pharmacy, Medical University of Lublin, 8B Jaczewskiego Str., 20-090 Lublin, Poland; dorota.natorska-chomicka@umlub.edu.pl (D.N.-C.); monika.gawronska-grzywacz@umlub.edu.pl (M.G.-G.); mariola.herbet@umlub.edu.pl (M.H.); iwona.piatkowska-chmiel@umlub.edu.pl (I.P.-C.); magdalena.iwan@umlub.edu.pl (M.I.); jaroslaw.dudka@umlub.pl (J.D.); 2 Chair and Department of Organic Chemistry, Faculty of Pharmacy, Medical University of Lublin, 4A Chodźki Str., 20-093 Lublin, Poland; pawelpatrejko@gmail.com

**Keywords:** hydrazide–hydrazone, bioactivity, antitumor activity, glioblastoma

## Abstract

Gliomas are highly aggressive tumors of the nervous system and remain difficult to treat with currently available therapeutic approaches. Due to their poor prognosis and resistance to standard treatments, there is a growing need for novel strategies, including therapies based on the combined use of radiotherapy and chemotherapy. The aim of this study was to evaluate the potential of newly synthesized hydrazide–hydrazones to sensitize glioblastoma tumor cells to X-ray irradiation. Two novel hydrazide–hydrazones of 5-bromo-2-iodobenzoic acid (**3** and **4**) were synthesized on the basis of condensation reaction. The chemical structure of obtained compounds was established with the use of IR, ^1^H NMR and ^13^C NMR spectroscopy. In vitro biological studies demonstrated that the radiosensitizing effect of the tested hydrazide–hydrazones was strongly dependent on both compound concentration and glioblastoma cell line. In LN-229 cells, compound **3** at 164 μM combined with 2 Gy irradiation reduced cell viability by 65% and increased the subG1 population, indicating enhanced cytotoxicity and induction of cell death. Similarly, compound **4** at 242 μM combined with 2–5 Gy irradiation decreased LN-229 cell viability by more than 50% and promoted cell cycle arrest and apoptosis, whereas both compounds showed limited or even proliferative effects in U-87MG cells, highlighting the importance of tumor-specific biological characteristics in determining treatment response.

## 1. Introduction

Glioblastoma multiforme (GBM) is a highly aggressive and invasive type of glioma classified as a primary central nervous system tumor. It is also the most common and malignant form, accounting for about 47% of all gliomas. These neoplasms comprise 80% of the malignant tumors originating in the brain and central nervous system (CNS) [1]. The classification of gliomas, which are not a homogenous concept in terms of histology, relies on the criteria established by the World Health Organization (WHO) grading system. Gliomas include ependymomas, astrocytomas (inter alia GBM), oligodendrogliomas, and mixed gliomas. Thus, among this group it is possible to distinguish between benign tumors, such as ependymomas, and very aggressive and fatal tumors, such as GBM, which is WHO grade IV cancer [2,3]. According to WHO criteria the predominant types of glioma in adults are astrocytoma and oligodendroglioma [4]. The highest grade IV refers to cytologically malignant and mitotically active lesions. Glioblastoma multiforme is associated with a high mortality rate [5]. The incidence is 5 cases per 100,000 people per year. In adults, the incidence increases with age, with a peak incidence in the 5th and 6th decades of life. Key pathways concerning glioma biology include the activation of growth factor receptor tyrosine kinases, which trigger signaling cascades such as the MAP kinase pathway or PI3K signaling. Additionally, there is the inhibition of apoptosis mediated by p53, control over the cell cycle, stimulation of angiogenesis through VEGF signaling, and mechanisms facilitating invasion [6].

There are few treatment choices for glioma. The initial step in treating all forms of glioma usually involves surgically removing the tumor, which may be repeated if feasible in cases of disease progression. Typically, surgical resection is combined with radiotherapy and adjuvant chemotherapy with temozolomide and carmustin. In case of metastasis alkylating chemotherapy with lomustine is used. Despite the complex treatment, recurrences are very common and treated with bevacizumab, an anti-VEGF monoclonal antibody [2,7,8,9]. Despite continuous medical developments and the search for new effective treatments, GBM is still a cancer with very poor prognosis. Most patients survive only several months after diagnosis, dying most often after 15 months of fighting the disease. Only 3% to 8% of glioblastoma patients live longer than three years, whereas only 5% reach the 5-year survival milestone [2,10].

Therefore, there is an urgent need to develop novel therapeutic strategies that can effectively target GBM cells and improve patient outcomes. Unfortunately, therapeutic strategies often prove ineffective due to the heterogeneity of tumors and the resistance they develop against cytotoxic alkylating agents [11]. Clinical practice has shown that combining radiation therapy with chemotherapy can significantly improve the survival rate and quality of life for GBM patients [2,7,8,9]. In addition, several adjuvant radiobiological approaches have been proposed to improve the therapeutic ratio of radiotherapy. Among them, radioenhancers have attracted considerable interest because they can increase radiation effects within the tumor while potentially limiting damage to the surrounding healthy brain parenchyma [12,13]. Such strategies are particularly relevant in neuro-oncology, where preserving normal tissue function is crucial. In contrast, radiosensitizers are designed to increase the susceptibility of tumor cells to ionizing radiation, thereby enhancing radiation-induced cell killing. X-rays are a form of high-energy electromagnetic radiation that can penetrate tissues and cause ionization of atoms and molecules. In the context of cancer treatment, X-rays induce a variety of DNA lesions, including single-strand breaks and the more cytotoxic double-strand breaks, either directly or indirectly through the generation of reactive oxygen species. The accumulation of unrepaired DNA damage may ultimately lead to cancer cell death [14]. Although X-rays are commonly used in the treatment of GBM, the efficacy of radiation therapy is limited by the intrinsic radioresistance of GBM cells, which is associated, among other factors, with their enhanced capacity to repair radiation-induced DNA damage [15]. Therefore, the development of novel radiosensitizing agents remains an important strategy to enhance the efficacy of radiotherapy and improve therapeutic outcomes.

New molecules presented in this study are hydrazide–hydrazones—a class of organic compounds that have attracted significant attention as potential radiosensitizers and chemotherapeutic agents for cancer treatment. They possess a variety of pharmacological properties, including antimicrobial, antioxidant, anti-inflammatory, and antitumor activities [16,17,18,19,20,21,22,23,24,25,26,27,28,29,30,31,32,33]. Moreover, some hydrazide–hydrazones have been shown to exhibit selective cytotoxicity towards cancer cells, making them promising candidates for targeted cancer therapy [22,23,24,25,26,27,28,29,30,31,32,33]. It was proved that stable metal complexes of hydrazones with Cu(II), Ni(II), Co(II), Pd(II), and Pt(II) ions demonstrated the ability to generate reactive oxygen species (ROS), which lead to DNA damage and the induction of apoptosis in cancer cells. Researchers have shown that these derivatives with a hydrazone moiety can also interact with enzymes involved in proliferation, such as protein kinases and metalloproteinases [34]. Many studies indicated that hydrazide–hydrazone derivatives significantly inhibited proliferation of human cancer cell lines derived from breast cancers, the most frequently diagnosed among women (MCF-7, MDA-MB-231), and lung cancers in men (NCI-H460, A549), but also colon cancers (COLO 205, HCT 15), ovary cancer (SK-OV-3), cervical cancer (HeLa), gastric cancer (BGC-823), liver cancer (HepG2), renal cancer (769-P), leukemia (Jurkat, BV-173), and central nervous system tumors (SF-268, SH-SY-5Y) [22,23,24,25,26,27,28,29,30,31,32,33,35,36].

In our previous published research, we have tested other hydrazide–hydrazones of 5-bromo-2-iodobenzoic acid against a renal adenocarcinoma cell line (769-P) and hepatocellular carcinoma cell line (HepG2). All compounds inhibited cancer cell proliferation, however, a molecule with a 2-chlorophenyl group proved to be the most cytotoxic and selective against the abovementioned cancer lines [31]. Our research team also described a strong antiproliferative effect of another series of hydrazide–hydrazones with a 3-hydroxy-2-naphthoic moiety towards the 769-P cancer line [32]. In addition, the results published in the scientific literature clearly indicated various hydrazide–hydrazones that decreased the viability of glioma cancer cells [37,38]. All the aforementioned studies encouraged us to analyze anticancer activity of two novel hydrazide–hydrazones of 5-bromo-2-iodobenzoic acid towards glioblastoma cell lines (U-87MG and LN-229). The research question explored in this study was therefore to related to the quantitative and qualitative evaluation of newly synthesized hydrazide–hydrazones to serve as radiosensitizers for glioblastoma tumor cells in response to X-ray irradiation.

## 2. Results

### 2.1. Chemistry

**Scheme 1:**
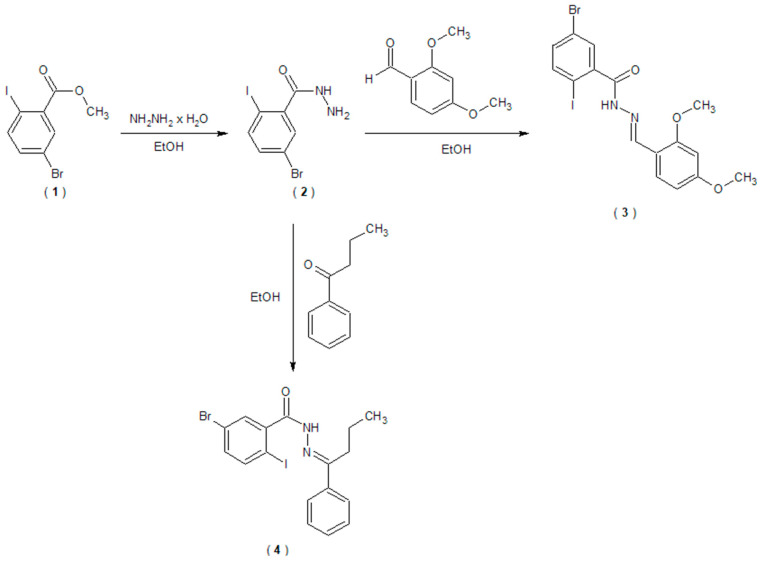
The route to the synthesis of hydrazide–hydrazones of 5-bromo-2-iodobenzoic acid.

In this research novel hydrazide–hydrazones (**3** and **4**) were synthesized by the condensation reaction of 5-bromo-2-iodobenzoic acid hydrazide (**2**) with an appropriate aldehyde or ketone. The synthesis was performed according to the procedure described earlier by our research group [31] and presented in Scheme 1. The chemical structure of obtained hydrazide–hydrazones was confirmed by the analysis of the IR, ^1^H NMR and ^13^C NMR spectra. In both the ^1^H NMR and ^13^C NMR spectra we found characteristic signals typical for chemical structure of hydrazide–hydrazones [16,17].

### 2.2. Biological Assays

**Figure 1:**
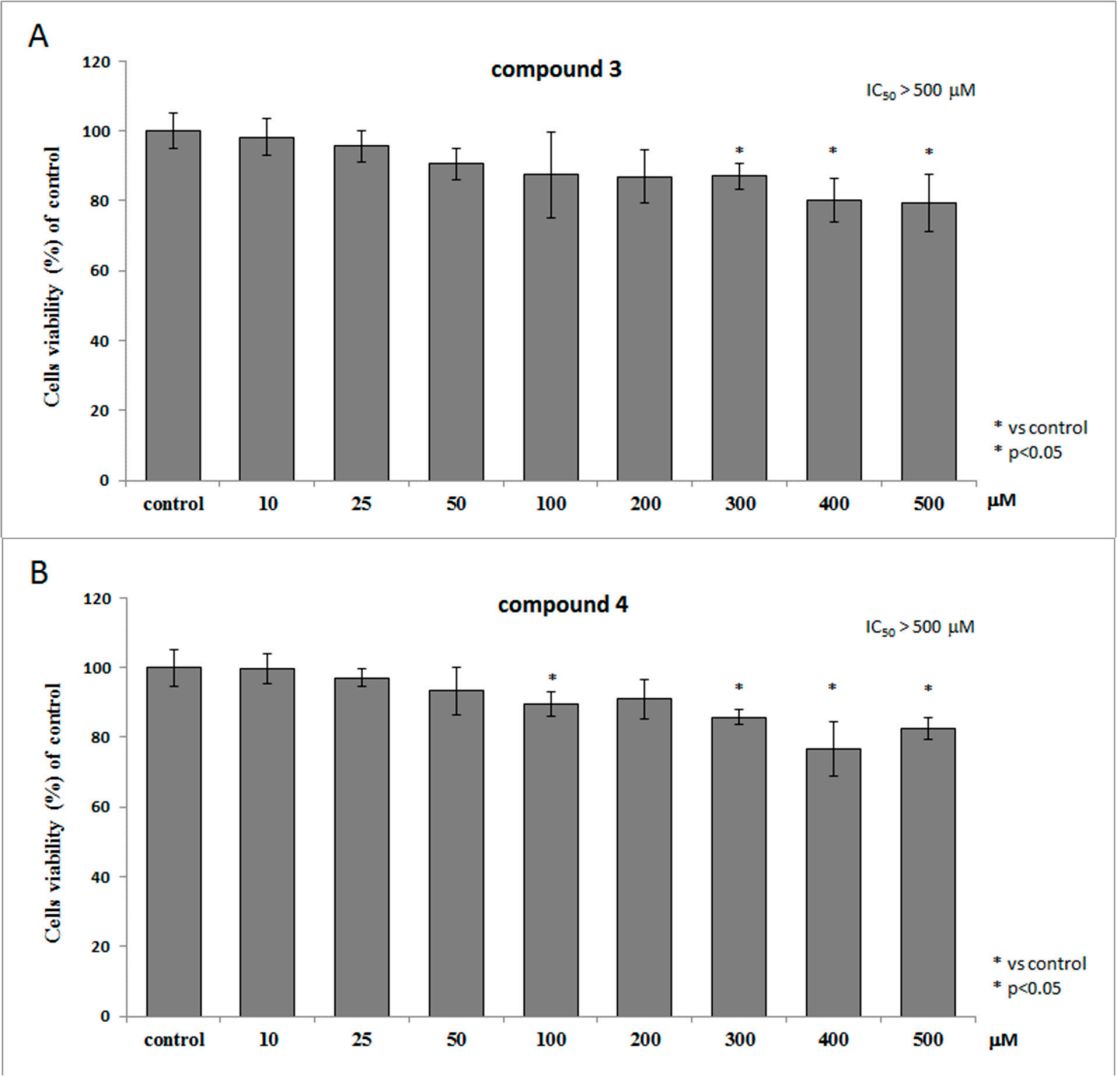
Viability of BJ fibroblast cell line after 24 h of incubation with compounds **3** (**A**) or **4** (**B**) determined by MTT assay (concentration 10–500 μM). The results were calculated as % of control cell viability which were defined as 100%. The experiments were performed in triplicate and the values were presented as mean ± SEM.

The newly synthesized hydrazide–hydrazones (compounds **3** and **4**) were tested for biological activity using in vitro methods. The cytotoxicity of the compounds **3** and **4** against normal cells was tested on BJ human fibroblasts using the MTT assay. After 24 h of incubation, slight toxicity of the tested compounds to BJ cells was noted. An insignificant decrease in cell viability was observed only at the highest concentrations (Figure 1A,B). The IC_50_ value of compounds **3** and **4** could not be determined.

In this study, the cytotoxic effect of compounds **3** and **4** (IC_10_, IC_25_ or IC_50_) alone and in combination with X-ray (2 Gy or 5 Gy) was examined on LN-229 and U-87MG glioblastoma cells. A dose-dependent decrease for a single compound was found in the cell viability of all tested glioma cell lines (Table 1). The estimated IC_50_ values for compound **3** for LN-229 and U-87MG were 164 and 181 μM, respectively. While for compound **4**, the IC_50_ values were calculated as 242 μM for line LN-229 and 171 μM for U-87MG cells.

**Figure 2:**
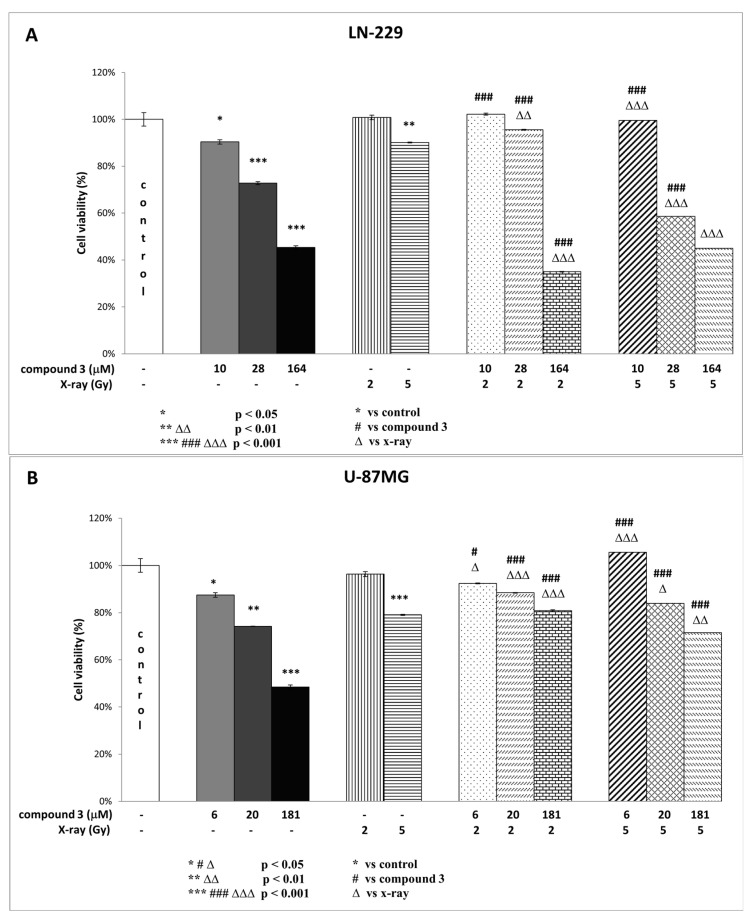
Effect of compound **3** and X-ray on the viability of LN-229 cells (**A**) and U-87MG cell line (**B**) after 24 h incubation with different concentrations of drugs, determined by MTT assay. The results were calculated as % of control cell viability which were defined as 100%. The experiments were performed in triplicate and the values were presented as mean ± SEM.

**Figure 3:**
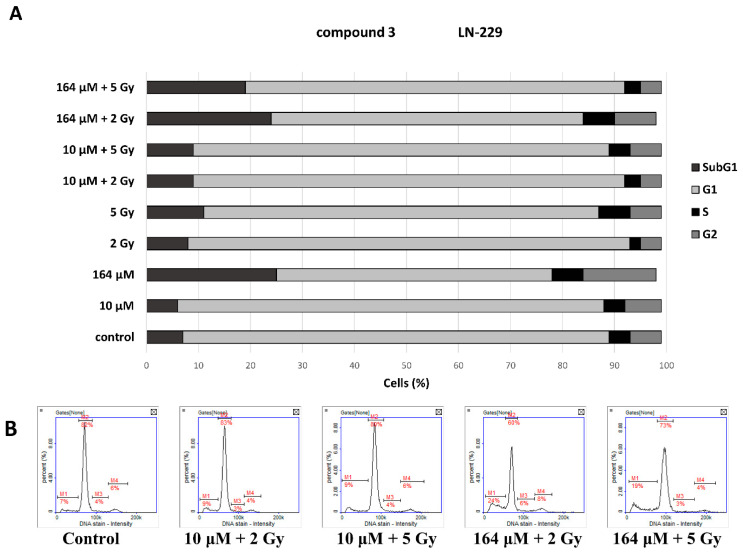
Cell cycle analysis by flow cytometry. The LN-229 cells were incubated for 48 h with compound **3**, after 24 h of incubation cells were irradiated with X-ray. Both (**A**) the bars and (**B**) the histograms present the distribution of cells in sub-G1, G1, S and G2 phases of the cell cycle.

Own research conducted on LN-229 glioma cells with the simultaneous use of compound **3** and X-rays showed significant changes in the viability of cancer cells (Figure 2A). The most effective combination was the concentration of 164 μM and radiation at a dose of 2 Gy, as it caused the greatest reduction in cell viability (by 65%) compared to radiation alone. At the same time, a significant increase in the number of cells in the sub-G1 phase and a decrease in the G1 phase were observed (Figure 3), which may indicate a cytotoxic effect of this combination, i.e., stopping the cell cycle in its early phases and inhibiting cell proliferation. The application of 10 μM and 28 μM concentrations of hydrazide–hydrazone **3** to LN-229 glioma in combination with X-rays resulted in an increase in cell viability, with no significant changes in the cell cycle. Low concentrations of compound **3** had too small a toxic effect on cancer cells to inhibit division and led to apoptosis but may be a signal stimulating their growth and proliferation.

**Figure 4:**
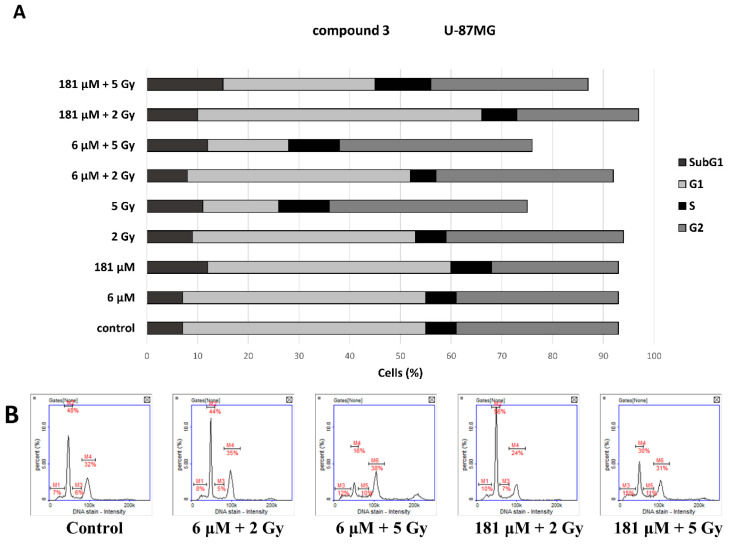
Cell cycle analysis by flow cytometry. The U-87MG cells were incubated for 48 h with compound **3**, after 24 h of incubation cells were irradiated with X-ray. Both (**A**) the bars and (**B**) the histograms present the distribution of cells in sub-G1, G1, S and G2 phases of the cell cycle.

Interesting results were obtained for the U-87MG cells line after incubation with substance **3** and after exposure to X-rays (Figure 2B). It was observed that the combination of these two factors very strongly stimulates glioblastoma cells to proliferate and the viability of cancer cells increased proportionally to the concentration of the compound **3**, as well as to the dose of radiation. This is a disadvantage of the use of this combination in the anticancer therapy of glioblastoma in vivo. While in cell cycle analysis, cell arrest was noted mainly in the sub-G1 phase (Figure 4). More detailed research on this subject should be carried out, considering the phenotypic and genotypic differences between LN-229 and U-87MG lines, in order to explain and indicate the reasons for such significant differences in the effects of tested compounds and X-rays on each of these cellular lines of glioblastoma.

**Figure 5:**
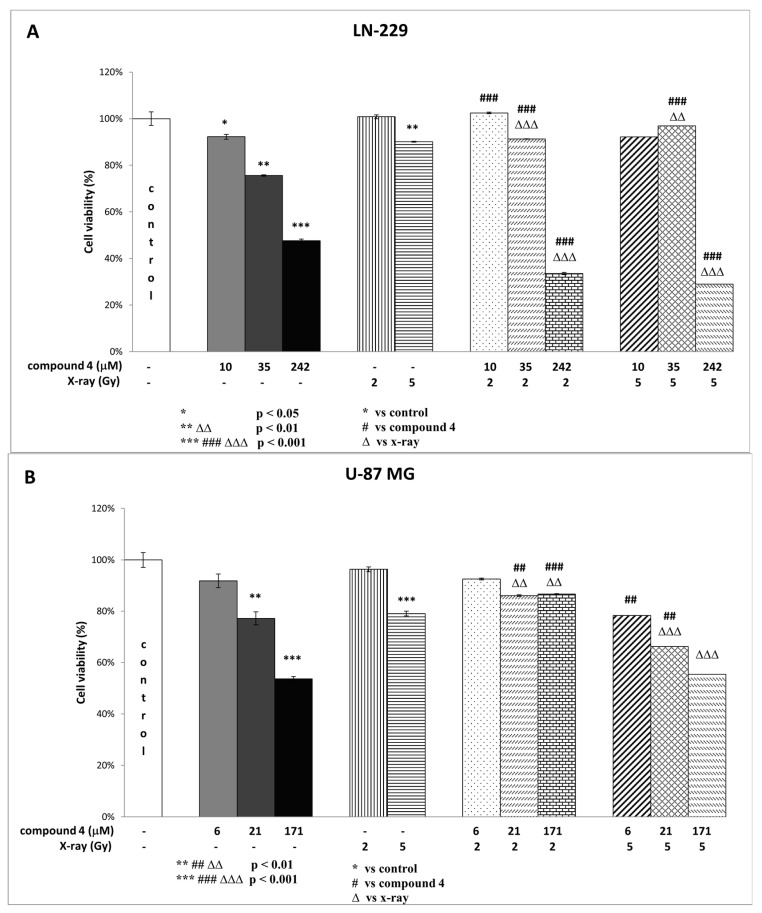
Effect of compound **4** and X-ray on the viability of LN-229 cells (**A**) and U-87MG cell line (**B**) after 24 h incubation with different concentrations of drugs, determined by MTT assay. The results were calculated as % of control cell viability which were defined as 100%. The experiments were performed in triplicate and the values were presented as mean ± SEM.

**Figure 6:**
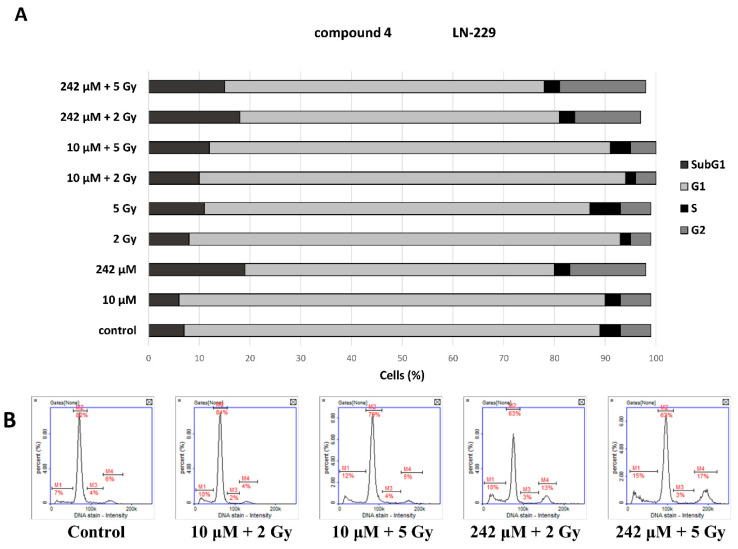
Cell cycle analysis by flow cytometry. The LN-229 cells were incubated for 48 h with compound **4**, after 24 h of incubation cells were irradiated with X-ray. Both (**A**) the bars and (**B**) the histograms present the distribution of cells in sub-G1, G1, S and G2 phases of the cell cycle.

The MTT test results for the combination of the compound **4** at a concentration of 242 μM with X-ray radiation at a dose of 2 Gy and 5 Gy proved to be sufficiently effective in the treatment of LN-229 glioma (Figure 5A). Cell viability had decreased by more than half, and there was a significant increase in cell population in the sub-G1 and G2 phases (Figure 6). These results indicate that cell division is stopped and cells are destroyed by apoptosis. Therefore, it can be assumed that compound **4** sensitizes the LN-229 tumor cells to X-radiation. As in the case of the compound **3**, after incubation of the cells with the lowest concentration of substance **4** used in the study (10 μM) and after irradiation with X-radiation in both doses, an unfavorable increase in tumor cell viability was noted.

**Figure 7:**
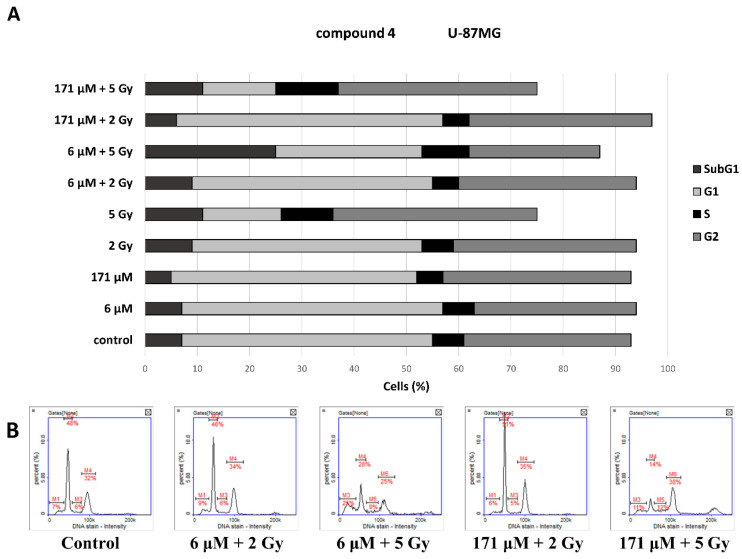
Cell cycle analysis by flow cytometry. The U-87MG cells were incubated for 48 h with compound **4**, after 24 h of incubation cells were irradiated with X-ray. Both (**A**) the bars and (**B**) the histograms present the distribution of cells in sub-G1, G1, S and G2 phases of the cell cycle.

In the case of the combination of compound **4** at a concentration of 6 μM and X-rays at a dose of 5 Gy, the viability of U-87MG glioma cells was reduced by only 13.5% (Figure 5B). However, the results of cell cycle studies indicate a clear inhibition of division in the sub-G1 phase (Figure 7). A similar result was noted after increasing the concentration to 171 μM, but without significant changes in the individual phases of the cell cycle compared to the control.

**Figure 8:**
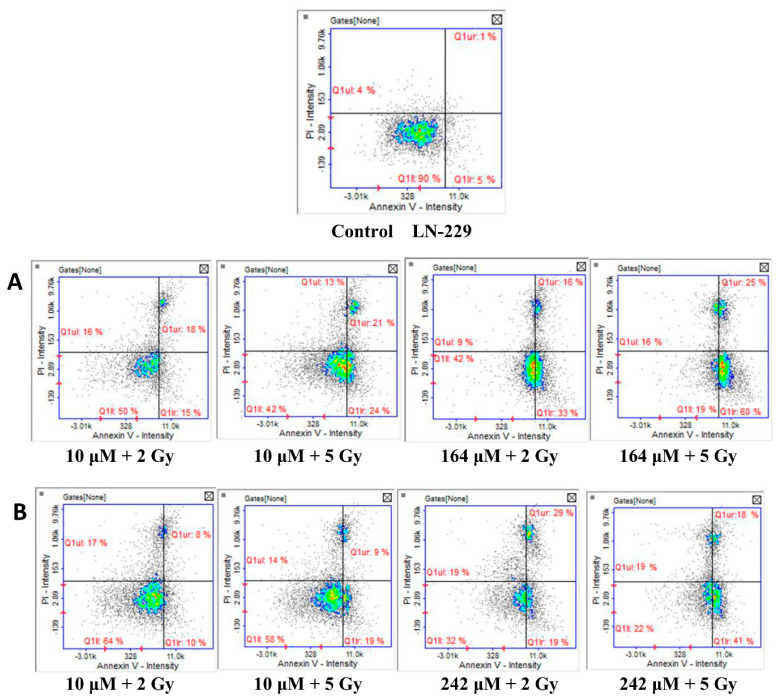
Effect of compound **3** (**A**) or **4** (**B**) and X-ray radiation on apoptosis in LN-229 cell line. Representative Annexin V/PI scatter plots obtained using the NucleoCounter^®^ NC-3000™ image cytometer are shown. The analyzed cell population was divided by gating into the following subpopulations: Q1ll—living cells, Q1lr—early apoptotic cells, Q1ur—late apoptotic cells, and Q1ul—dead (necrotic) cells. The pseudocolor scale represents the local density of analyzed cells, with cooler colors indicating lower cell density and warmer colors indicating higher cell density.

**Figure 9:**
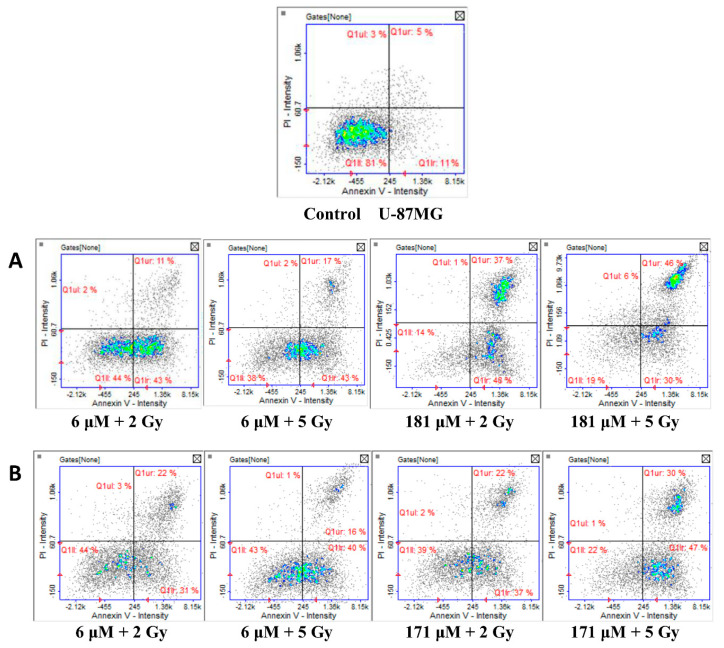
Effect of compound **3** (**A**) or **4** (**B**) and X-ray radiation on apoptosis in U-87MG cell line. Representative Annexin V/PI scatter plots obtained using the NucleoCounter^®^ NC-3000™ image cytometer are shown. The analyzed cell population was divided by gating into the following subpopulations: Q1ll—living cells, Q1lr—early apoptotic cells, Q1ur—late apoptotic cells, and Q1ul—dead (necrotic) cells. The pseudocolor scale represents the local density of analyzed cells, with cooler colors indicating lower cell density and warmer colors indicating higher cell density.

Additionally, apoptosis/necrosis analysis after incubation with compounds **3** or **4** with image cytometry revealed that both hydrazide–hydrazones in combination with X-rays increase the percentage of early- and late-apoptotic and necrotic cells compared to the control. The effect is dependent on the concentration used. The largest number of cells in the necrotic phase was observed after incubation of the LN-229 glioblastoma cell line (Figure 8). While in the case of the U-87MG cells, the cells underwent only early or late apoptosis (Figure 9).

## 3. Discussion

### 3.1. Chemistry

Application of ^1^H NMR and ^13^C NMR spectroscopy enabled confirmation of the chemical structure of the synthesized compounds and correctness of the condensation reaction. According to previously published research these methods are sufficient to confirm chemical structure of compounds from this group [16,17].

In the ^1^H NMR spectra of compound **3** we found characteristic singlet signals for this class of compounds. These included a singlet signal at δ 8.83 ppm which corresponded to the proton of the =CH group and a singlet signal for the proton of the amino group NH at δ 11.72 ppm. In the ^13^C NMR for this compound we registered a characteristic signal for the carbon atom of the =CH group at δ 142.2 ppm.

In the case of the ^1^H NMR of compound **4** we did not find a signal for the proton of =CH because this reaction was performed with a ketone, not with an aldehyde, as for compound **3**. We have found a singlet signal for the proton of the NH group at δ 10.98 ppm. In the ^13^C NMR we found a characteristic signal for the tertiary carbon atom at δ 151.1 ppm.

Signals for other atoms and fragments in the ^1^H NMR and ^13^C NMR for compounds **3** and **4** were found at expected values of chemical shift. Signals present on the IR spectra of compounds **3** and **4** were found at expected values.

### 3.2. Biological Assays

Based on the results of our study, it may be highlighted that tested hydrazide–hydrazones of 5-bromo-2-iodobenzoic acid (**3** and **4**) at the highest concentration tested sensitize the LN-229 glioblastoma cells to X-radiation, resulting in a decrease in cell viability, disruption of the cell cycle and apoptosis. Viswanathan et al. [38] conducted similar studies on LN-229 and U-87MG glioma cells using their synthesized hydrazones. They showed that many derivatives significantly reduced the viability of cancer cells and inhibited their proliferation. This proves the key role played here by the chemical structure of these compounds. Aryl derivatives of hydrazones show significantly greater anticancer activity, and the IC_50_ values for these substances are comparable to those of cis-platinum.

The results of this study are promising and suggest that the combination of hydrazide–hydrazones and X-ray radiation therapy may represent a new and effective treatment option for glioblastoma. Further research is needed to fully understand the mechanisms of action of hydrazide–hydrazones and to determine the optimal dosing and administration schedules for this promising therapy.

Several studies have investigated the effects of X-rays on GBM cells and their underlying mechanisms. For instance, research [39,40] has shown that X-rays can induce DNA damage and apoptosis in GBM cells by activating the ATM/Chk2 signaling pathway. The authors also found that the expression of p53, a tumor suppressor gene, was upregulated in response to X-ray exposure, indicating that p53-mediated apoptosis may contribute to the cytotoxic effects of X-rays on GBM cells.

In addition, radiation exposure can modulate the expression of microRNAs that regulate tumor cell survival. For example, miR-204 has been shown to suppress glioblastoma cell growth by targeting the antiapoptotic protein BCL-2, thereby promoting apoptosis [41,42].

Despite the potential benefits of radiation therapy, its use is limited by the potential side effects on healthy tissues and organs. For instance, exposure to ionizing radiation can induce oxidative stress, DNA damage, and inflammation in normal tissues, leading to tissue injury and dysfunction. Therefore, there is an urgent need to identify novel radiation sensitizers that can enhance the efficacy of radiation therapy while reducing its side effects.

## 4. Materials and Methods

### 4.1. Chemistry

#### 4.1.1. General Information

Reagents and solvents were purchased from Sigma-Aldrich (Munich, Germany) and Merck Co. (Darmstadt, Germany) and used without further purification. Melting points were measured on Fisher-Johns blocks melting point apparatus (Fisher Scientific, Schwerte, Germany) and left uncorrected. The Nicolet 6700 FT-IR spectrophotometer (Thermo Scientific, Waltham, MA, USA) in ATR mode was used to register the IR spectra of synthesized compounds. The ^1^H NMR and ^13^C NMR spectra were recorded on the Bruker Avance 600 apparatus (Bruker BioSpin GmbH, Rheinstetten, Germany) in DMSO-*d*_6_ with TMS as the internal standard. Chemical shifts are reported in ppm (δ) with the use of TMS as the standard reference. The coupling constants (*J*) are given in Hertz. The progress of the reaction and purity of obtained compounds were monitored by TLC using precoated aluminum sheet 60 F254 plates (Merck Co., Rahway, NJ, USA) in a CHCl_3_/C_2_H_5_OH (10:1, *v*/*v*) solvent system. The spots were detected by exposure to a UV lamp at 254 nm. The elemental analysis of obtained compounds was carried out with the AMZ 851 CHX analyzer (PG, Gdańsk, Poland). The results of elemental analysis (C, H, N) were within ± 0.4% of the calculated values.

#### 4.1.2. Synthesis of Novel Compounds

**The synthesis of 5-bromo-2-iodobenzoic acid hydrazide (2)**

The synthesis was performed with the use of methyl 5-bromo-2-iodobenzoate (**1**) according to the procedure reported earlier by our group [31].

5-bromo-2-iodobenzhydrazide (**2**)

CAS Registry Number: 1023146-61-3. Yield: 78%; M.p.: 98 °C. The physico-chemical properties of hydrazide of 5-bromo-2-iodobenzoic acid are consistent with those reported earlier by our group [31].

**The synthesis of hydrazide–hydrazones of 5-bromo-2-iodobenzoic acid (3, 4)**

The hydrazide–hydrazones of 5-bromo-2-iodobenzoic acid were synthesized with the use of the same method which we described earlier [31]. First, 0.001 mole of 5-bromo-2-iodobenzoic acid hydrazide (**2**) was dissolved in 5 mL of ethanol (96%). Then 0.0011 mole of appropriate aldehyde: 2,4-dimethoxybenzaldehyde (**3**) or ketone: 1-phenylbutan-1-one (**4**) was added and the mixture was heated under reflux for 3 h. After that the solution was allowed to cool and precipitate which formed was filtered off and re-crystallized from ethanol.

5-bromo-*N*-[(2,4-dimethoxyphenyl)methylidene]-2-iodobenzohydrazide (**3**)

Yield: 86%; M.p.: 146–148 °C. IR (cm^−1^): 3181 (NH), 3064 (CH, arom.), 2947 (CH, aliph.), 1665 (C=O), 1610 (C=N); ^1^H NMR (600 MHz, DMSO-*d*_6_) δ (ppm) = 3.85 (s, 3H, CH_3_), 3.84 (s, 3H, CH_3_), 6.63–6.67 (m, 1H, ArH), 7.35–7.38 (d, 1H, ArH, *J* = 9 Hz), 7.43–7.44 (d, 1H, ArH, *J* = 9 Hz), 7.69–7.70 (d, 1H, ArH, *J* = 3 Hz), 7.82–7.84 (d, 1H, ArH, *J* = 6 Hz), 7.87–7.89 (d, 1H, ArH, *J* = 6 Hz), 8.83 (s, 1H, =CH), 11.72 (s, 1H, NH); ^13^C NMR (150 MHz, DMSO) δ (ppm) = 55.9 (OCH_3_), 56.3 (OCH_3_), 93.7, 100.3, 107.1, 116.2, 122.8, 129.7, 131.2, 133.3, 140.5 (9C_ar_), 142.2 (=CH), 160.9, 162.8, 163.8 (3C_ar_), 169.4 (C=O); analysis for C_16_H_14_BrIN_2_O_3_ (489.10): calculated: C: 39.29%, H: 2.89%, N: 5.73%; found: C: 39.34%, H: 2.91%, N: 5.78%.

5-bromo-2-iodo-*N*-[(1-phenylbutylidene]benzohydrazide (**4**)

Yield: 34%; M.p.: 178–180 °C. IR (cm^−1^): 3208 (NH), 3051 (CH, arom.), 2950, 2907 (CH, aliph.), 1648 (C=O), 1605 (C=N); ^1^H NMR (600 MHz, DMSO-*d*_6_) δ (ppm) = 1.01–1.06 (t, 3H, CH_3_, *J* = 9 Hz, *J* = 6 Hz), 2.20–2.34 (m, 2H, CH_2_), 3.41–3.45 (t, 2H, CH_2_, *J* = 6 Hz), 7.26–7.30 (m, 1H, ArH), 7.34–7.39 (m, 1H, ArH), 7.40–7.45 (m, 2H, ArH), 7.51–7.52 (d, 1H, ArH, *J* = 3 Hz), 7.67–7.68 (d, 1H, ArH, *J* = 3 Hz), 7.79–7.85 (m, 1H, ArH), 7.89–7.93 (m, 1H, ArH), 10.98 (s, 1H, NH); ^13^C NMR (150 MHz, DMSO) δ (ppm) = 13.6 (CH_3_), 20.4 (CH_2_), 32.2 (CH_2_), 91.2, 122.7, 125.8, 128.4, 128.5, 131.2, 133.3, 140.5, 142.2 (12C_ar_), 151.1 (C_IV_), 163.7 (C=O); analysis for C_17_H_16_BrIN_2_O (471.13): calculated: C: 43.34%, H: 3.42%, N: 5.95%; found: C: 43.39%, H: 3.39%, N: 5.99%.

### 4.2. Cell Culture

The present study was performed using two human glioblastoma cell lines: U-87MG (catalogue no. HTB-14™) and LN-229 (catalos no. CRL-2611™), and fibroblast cell line BJ (catalogue no. CRL-2522™), which was used as a normal reference cell line, obtained from the American Type Culture Collection (Manassas, VA, USA). The U-87MG and BJ cell lines were cultured in Eagle’s minimal essential medium (EMEM) and the LN-229 cell line in Dulbecco’s modified Eagle’s medium (DMEM) supplemented with 10% fetal bovine serum (FBS) and antibiotics: 100 U/mL penicillin, 100 μg/mL streptomycin and 2.5 μg/mL amphotericin B (PAN-Biotech GmbH, Aidenbach, Germany). Cells were grown in a humidified incubator at 37 °C and 5% CO_2_ atmosphere in 75 cm^2^ tissue culture flasks. Before the experiment, the cells were trypsinized (0.25% trypsin/2.21 nM EDTA) (PAN-Biotech GmbH, Aidenbach, Germany) and cells were seeded into 6-well or 96-well plates (depending on the assay) at a density of 1 × 10^5^ cells/mL. The prepared plates were incubated for 24 h in order to achieve cell adhesion.

### 4.3. MTT Assay/Cell Viability Assay/Cell Growth Inhibitory Assay

The MTT assay (thiazolyl blue tetrazolium bromide, Sigma-Aldrich, Merck KGaA, Darmstadt, Germany) was used to assess the cell growth inhibitory effect of the new compounds 3 and 4, based on DB-ALM protocol No. 17 (DataBase service on Alternative Methods), produced by the European Union Reference Laboratory on Alternatives to Animal Testing (EURL ECVAM) [43]. The cell viability was determined in a mitochondrial-dependent reaction: reduction of yellow tetrazolium salt to purple formazan crystals. The results were expressed as the percentage of treated cells in relation to untreated control cells, considered as 100%. Compounds **3** and **4** were dissolved in DMSO and next diluted with an appropriate culture medium (final DMSO concentration was <0.5%). The solutions were prepared *ex tempore*. Cells were cultured in 96-well plates with the different concentrations (10–500 μM) of the tested compounds **3** or **4** for 24 h. At the end of treatment, 10 µL MTT (final concentration 5 mg/mL) was added to each well of a microplate and incubated for 3 h at 37 °C. Afterwards, the formazan crystals produced were dissolved with DMSO and then the absorbance of each well was measured at 550 nm using an ELx808IU automated absorbance microplate reader (BioTek Instruments, Inc., Winooski, VT, USA). Based on the MTT results obtained, the IC_10_, IC_25_ and IC_50_ values of each tested compound were calculated from the concentration dose–response curves. All the experiments were performed in triplicate.

### 4.4. X-Ray Radiation

The RS 2000 X-ray biological irradiator (X Rad-Source RS 2000 Biological System, Rad Source Technologies, Inc., Suwanee, GA, USA) working at 160 kV and 25 mA was used. Cells were seeded into 6-well or 96-well plates (depending on the assay) at a density of 1 × 10^5^ cells/mL. After 48 h of incubation, cells were irradiated with doses of 2 and 5 Gy corresponding to irradiation times of 30 and 75 s.

### 4.5. Treatment of Cell Culture

The cells of gliomas were seeded into 6-well or 96-well plates (depending on the assay) at the density 1 × 10^5^ cells/mL. After 24 h of incubation, cells were treated with the IC_10_, IC_25_ or IC_50_ concentration of compounds **3** and **4** (Table 2). After another 24 h, cells were irradiated with 2 or 5 Gy X-ray.

### 4.6. Cell Cycle

The impact of the novel hydrazide–hydrazones **3** and **4** with or without X-rays on the cell cycle was analyzed by the image cytometer NucleoCounter NC-3000 (ChemoMetec, Allerød, Denmark), according to the NC-3000 Two-step Cell Cycle Analysis Protocol. The cells were seeded into 6-well plates at a density of 1 × 10^5^ cells/mL. After the incubation of cells with newly synthesized compounds and after irradiation with X-rays (according to Section 4.5. Treatment of Cell Culture), cells were trypsinized and harvested by centrifugation at 400× *g* for 5 min. at room temperature. Next, the precipitate of cells was washed once with PBS, resuspended in 250 μL of lysis buffer (Solution 10) supplemented with 10 μg/mL DAPI and incubated at 37 °C for 5 min. Subsequently, 250 μL of stabilization buffer (Solution 11) was added, 30 μL of each of the obtained cell suspensions was applied to the NC-Slide A2™ and analyzed in the NucleoCounter NC-3000. Cellular fluorescence was measured at 365 nm and quantified. Markers in the displayed histograms of DNA were used to demarcate cells in the different cell cycle stages. The results are presented as the percentages of the cells in the different cell cycle phases: sub-G1, G1/G0, S or G2/M.

### 4.7. Apoptosis Detection/Annexin V Assay

The assessment of apoptosis was performed on the basis of the protocol Annexin V Assay No. 3017 using the NucleoCounter NC-3000 system. The cells were seeded into 6-well plates. After the incubation of cells with compound **3** or **4** and after irradiation with X-rays (according to Section 4.5. Treatment of Cell Culture), the cells were harvested by centrifugation at 400× *g* for 5 min. at room temperature and were washed once with PBS. Subsequently, the cell pellet was resuspended in 100 μL of Annexin V binding buffer. In the next step, 2 μL of Annexin V-CF488A conjugate and 2 μL of solution containing Hoechst 33342 were added to the glioma cells and mixed by pipetting. The samples were incubated at 37 °C for 15 min. After incubation, the stained cells were centrifuged and resuspended again in 100 μL of Annexin V binding buffer supplemented with 10 μg/mL of solution containing PI. Then, 30 μL of each of the obtained cell suspensions was applied to the NC-Slide A2™ and analyzed in the NucleoCounter NC-3000. This assay determined cell death via apoptotic or necrotic pathways. From the scatterplots showing the fluorescence intensity of Annexin V-CF488A and PI, the ratio of apoptotic and late-apoptotic/necrotic cells could be determined.

### 4.8. Statistical Analysis

All data were analyzed using Statistica software, version 13 (StatSoft, Krakow, Poland). The results were presented as the mean ± SEM. The statistical comparisons between groups were performed by ANOVA, followed by a Bonferroni post hoc test. A *p* value under 0.5 was considered statistically significant.

## 5. Conclusions

In conclusion, radiotherapy with X-rays combined with compounds possessing hydrazide–hydrazone groups represents a promising therapeutic strategy for the treatment of glioblastoma. While the use in clinical practice is still limited, the results obtained in preclinical studies suggest that it could be effective in overcoming the challenges associated with the treatment of glioblastoma. This research offers hope for patients with this devastating disease and underscores the importance of continued research into new and innovative therapies for cancer. However, future research should focus on the development of more precise and targeted approaches to deliver X-rays to the tumor and on the identification of more selective and less toxic hydrazide–hydrazones that can specifically target cancer cells.

## Figures and Tables

**Table 1:** Antiproliferative activity of compounds **3** and **4** on glioma cell lines.

	LN-229			U-87MG		
	IC_10_ (μM)	IC_25_ (μM)	IC_50_ (μM)	IC_10_ (μM)	IC_25_ (μM)	IC_50_ (μM)
Compound **3**	10	28	164	6	20	181
Compound **4**	10	35	242	6	21	171

**Table 2:** Scheme for treating cells with a combination of compound 3 or 4 and X-rays.

LN-229						
Compound **3** + X-ray	IC_10_ + 2 Gy	IC_10_ + 5 Gy	IC_25_ + 2 Gy	IC_25_ + 5 Gy	IC_50_ + 2 Gy	IC_50_ + 5 Gy
Compound **4** + X-ray	IC_10_ + 2 Gy	IC_10_ + 5 Gy	IC_25_ + 2 Gy	IC_25_ + 5 Gy	IC_50_ + 2 Gy	IC_50_ + 5 Gy
U-87MG						
Compound **3** + X-ray	IC_10_ + 2 Gy	IC_10_ + 5 Gy	IC_25_ + 2 Gy	IC_25_ + 2 Gy	IC_50_ + 2 Gy	IC_50_ + 5 Gy
Compound **4** + X-ray	IC_10_ + 2 Gy	IC_10_ + 5 Gy	IC_25_ + 2 Gy	IC_25_ + 5 Gy	IC_50_ + 2 Gy	IC_50_ + 2 Gy

## Data Availability

The original contributions presented in this study are included in the article. Further inquiries can be directed to the corresponding author.
